# Cytoskeletal Arrest: An Anoxia Tolerance Mechanism

**DOI:** 10.3390/metabo11080561

**Published:** 2021-08-23

**Authors:** Alexander Myrka, Leslie Buck

**Affiliations:** 1 Department of Cell and Systems Biology, University of Toronto, Toronto, ON M5S 3G5, Canada; alex.myrka@mail.utoronto.ca; 2 Department of Ecology and Evolutionary Biology, University of Toronto, Toronto, ON M5S 3G5, Canada

**Keywords:** actin, tubulin, mitochondria, mitochondrial membrane potential, calcium, anoxia, temperature, western painted turtle, ROS, gasotransmitters

## Abstract

Polymerization of actin filaments and microtubules constitutes a ubiquitous demand for cellular adenosine-5′-triphosphate (ATP) and guanosine-5′-triphosphate (GTP). In anoxia-tolerant animals, ATP consumption is minimized during overwintering conditions, but little is known about the role of cell structure in anoxia tolerance. Studies of overwintering mammals have revealed that microtubule stability in neurites is reduced at low temperature, resulting in withdrawal of neurites and reduced abundance of excitatory synapses. Literature for turtles is consistent with a similar downregulation of peripheral cytoskeletal activity in brain and liver during anoxic overwintering. Downregulation of actin dynamics, as well as modification to microtubule organization, may play vital roles in facilitating anoxia tolerance. Mitochondrial calcium release occurs during anoxia in turtle neurons, and subsequent activation of calcium-binding proteins likely regulates cytoskeletal stability. Production of reactive oxygen species (ROS) formation can lead to catastrophic cytoskeletal damage during overwintering and ROS production can be regulated by the dynamics of mitochondrial interconnectivity. Therefore, suppression of ROS formation is likely an important aspect of cytoskeletal arrest. Furthermore, gasotransmitters can regulate ROS levels, as well as cytoskeletal contractility and rearrangement. In this review we will explore the energetic costs of cytoskeletal activity, the cellular mechanisms regulating it, and the potential for cytoskeletal arrest being an important mechanism permitting long-term anoxia survival in anoxia-tolerant species, such as the western painted turtle and goldfish.

## 1. The Anoxia-Tolerant Western Painted Turtle

Since the endosymbiotic origin of mitochondria [1], oxygen availability has been essential to eukaryote life, but animal species have repeatedly evolved to occupy niches that experience periods where oxygen availability is low (hypoxia), or absent entirely (anoxia; [2]). Research into how animals maintain hypoxic energy homeostasis was sparked in part by the diving behaviour of seals, which was studied by Scholander, Irving, and Grinnell in the 1940s [2,3], igniting interest in other anoxia-tolerant model organisms. Attention turned to diving turtles in the 1960s, when Jackson [4] showed, for the first time in a vertebrate species, a reversible metabolic suppression during anoxia. Whole animal calorimetry revealed that heat loss by anoxia-tolerant red-eared slider turtles (*Trachemys scripta elegans*) fell by 40% during four hours of anoxia and recovered with reoxygenation. Subsequently, the western painted turtle *Chrysemys picta belli* was ultimately determined to be the most anoxia-tolerant vertebrate tetrapod. This is likely the result of its northern overwintering range, which necessitates the ability to survive under ice-covered lakes and ponds for up to four months [5,6].

Studies on western painted turtles have focused on adenosine-5′-triphosphate (ATP) conservation in the brain and the reduction of ATP demand to match anoxic ATP supply (10% of normoxic ATP supply) through such mechanisms as ion channel arrest, spike arrest, and synaptic arrest (reviewed in [7]), as well as transcription and translation arrest [8,9]. Investigations have also targeted heart and liver (reviewed in [10]), with studies of non-excitable cells primarily limited to liver tissue. Adaptations of non-excitable cells to anoxia likely differ from those of excitable cells, as spike arrest is not a factor. Buck et al. [11] proposed isolated turtle hepatocytes as a model anoxia-tolerant primary cell system due to the relative homogeneity of cell type and size, large glycogen reserves, and role in whole animal anoxic metabolism. Research into hepatocytes has provided evidence for ion channel arrest as well as downregulation of glycolysis and biosynthetic processes such as protein turnover and urea synthesis (reviewed in [12]). To date, no studies have investigated the role of underlying structural processes in reducing anoxic ATP demand, and the phenotype of overwintering turtle cells remains unexplored. In this review we will discuss anoxia tolerance through the lens of the cytoskeleton and cellular structure and will use this foundation to build the “Cytoskeletal Arrest Hypothesis”. We have especially drawn on anoxia tolerance of the western painted turtle (painted turtle) and the more readily and widely studied red-eared slider (red-eared turtle) and have included implications for mitochondrial function.

## 2. Cytoskeletal Dynamics Consume ATP

**Figure 1:**
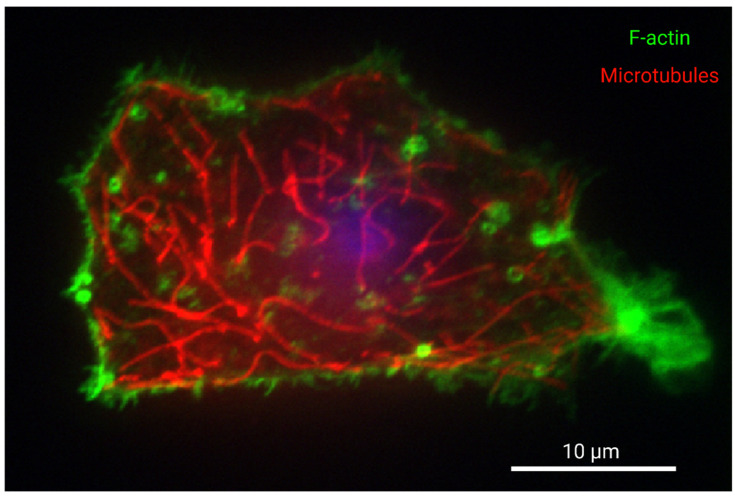
Organization of F-actin and microtubules in a cultured western painted turtle hepatocyte. Turtle hepatocytes were isolated as part of another study which was approved by the University of Toronto Animal Care Committee, and turtles were supplied by Niles Biological (Sacramento, CA, USA) and housed as described previously [44]. Hepatocytes were isolated as described by [11] and with sterile conditions, but with the addition of 1X Gibco MEM amino acids, 1X Gibco NEM amino acids, 1X Gibco MEM vitamin solution, 1X Pen-strep (Sigma Aldrich, St. Louis, MO, USA), and 200 μg/mL gentamicin sulphate (Bioshop) to the final media. Cells were cultured for 24 h at 15 °C on glass bottom dishes coated using 2.5 µg/mL purified fibronectin (MilliporeSigma, Burlington, MA, USA) in PBS. Cells were then permeabilized with 0.1% Triton X-100 in PBS for ten minutes, blocked with 7% BSA in PBST with 22.7 mg/mL glycine for one hour, and incubated overnight with mouse anti-α-Tubulin (DM1A) 3873 (Cell Signalling Technology, Danvers, MA, USA) with 1% BSA in PBS overnight at 4 °C. Next, cells were incubated with 1:1000 4′,6-diamidino-2-phenylindole DAPI, 1:200 Cy3 conjugated donkey AffiniPure fab fragment anti-mouse (Jackson ImmunoResearch, West Grove, PA, USA) and 1:100 Alexa Fluor 488 Phalloidin (Thermo Fisher Scientific, Waltham, MA, USA) in 1% BSA for one hour. The nucleus is presented in blue, tubulin in red, and F-actin in green. Samples were imaged using a Nikon eclipse Ti-2 spinning disk microscope and a Photometrics CoolSnap Myo CCD camera. Data was captured using NIS-Elements Viewer. Scale bar is 10 microns. The sample is focused on the site of attachment to the substrate. F-actin fluorescence staining intensity is observed most abundantly at the cortical cytoskeleton of the cell membrane, especially at adhesions and protrusions, while microtubules are well distributed throughout the cell. The nucleus is not in the focal plane, but its relative position is indicated by out-of-focus fluorescence. Image labels were created with BioRender.com.

Biological processes throughout a cell are organized and influenced by cytoskeletal structure, the energetic costs of which will be discussed here. Microtubule filaments (microtubules) are composed of tubulin monomers and, in addition to conferring mechanical stability, have key functions including long-distance-intracellular trafficking and organelle organization [13,14]. Filamentous actin (F-actin) is composed of actin monomers and is present in high abundance at the cortical cytoskeleton adjacent to the cell membrane, where it is more abundant than microtubules. At the cell periphery F-actin facilitates interactions between the cell, neighbouring cells, and the environment, such as cell spreading, migration, adhesion formation, and endocytosis [13,15,16,17]. While F-actin is highly abundant in the cell periphery [13,17], it is also involved in myriad functions throughout the cell such as force transduction through stress fibres [17], nuclear organization [18], mitochondrial fission [19], and short distance intracellular trafficking [20]. For reference, relative distribution of these two filaments in a cultured turtle hepatocyte is presented in Figure 1. F-actin interacts with the motor protein myosin to generate actomyosin contraction (cellular force generation resulting from the molecular motor light chain myosin acting on F-actin). Such contraction facilitates structural rearrangement and motility [21,22], as well as aforementioned cargo trafficking [20]. Intermediate filaments (IFs) confer mechanical stability and anchor assorted cell structures but, unlike microtubules and F-actin, they do not serve as tracks for motor proteins. They are composed of varying subunits depending on cell type and subcellular localization and they are particularly abundant in the perinuclear area, but are also found throughout the cell [13,23]. Actin and tubulin filaments are dynamic structures with rapid turnover that are modified near constantly by polymerization and depolymerization, referred to as “dynamic instability” for microtubules and “treadmilling” for F-actin, processes that, together with actomyosin contraction, are here referred to as “cytoskeletal dynamics”. The polymerization during these dynamics consumes ATP and guanosine-5′-triphosphate (GTP) for actin and tubulin, respectively [13]. The cost of this polymerization was calculated by Thuillier and Hauet [24] to be 16 ATP/37 nm for F-actin [24,25] and 16 GTP/8 nm for microtubules [24,26]. IFs also exhibit dynamic structure but have slower turnover and, crucially in the context of overwintering hypometabolism, IF assembly does not require hydrolysis of ATP or GTP [13,27]. Actomysin contraction, and intracellular trafficking along F-actin and microtubules by motor proteins, constitute additional ATP demands [28,29]. Observations of ATP consumption during such processes as cell spreading have established qualitatively that cytoskeletal actin and tubulin polymerization and actomyosin contraction are energetically expensive at the cellular level; the process of cell spreading reduces intracellular ATP by 22% [30,31]. True measurements of the ATP cost of background cytoskeletal polymerization in the cell do not exist because inhibition of these housekeeping processes also results in the inhibition of many other processes that depend upon cytoskeletal anchorage and dynamics, such as postsynaptic ion channel activity [32], glycolysis [33], and ion homeostasis and cell volume regulation [34], to name a few.

Whole body calculations of actinomyosin contraction (here referring to the contraction of sarcomeres in muscle tissue) estimate that it consumes 2–8% of the total oxygen consumption in a mammal, but this calculation does not include the contribution of cellular actin polymerization [28]. Given the technical inability to quantitatively isolate ATP consumption by actin or tubulin in a whole cell, estimates for ATP consumption have varied considerably, but several estimates are available for vertebrate brain. Estimations of the contribution of actin dynamics to neuronal ATP consumption have varied from less than 1% to greater than 50% (reviewed in [35]). One study estimated that neuronal synaptic vesicle turnover and associated actin dynamics utilized no more than 1% of synaptic ATP budget [35,36], but another found that inhibiting actin polymerization in embryonic chick neurons lowered cellular oxygen consumption by over 50% [37]. The higher measurement for embryonic chick neurons is no doubt due, in part, to high demand for cytoskeletal polymerization inherent to cell growth and division. Inhibition of neuronal actin polymerization also inhibits excitatory neurotransmitter receptors [32,38] and consequently the above value is likely further confounded by ATP use for neuronal action potential firing. Estimates for GTP consumption by tubulin dynamics are limited, but are generally less than those for actin dynamics, owing in part to the more rapid turnover of actin [39,40,41]. A theoretical calculation based on microtubule abundance in neurons estimated that GTP consumption by neuronal microtubule polymerization would only account for <1% of neuronal energy budget [35]. Another estimation, using inhibition of actin or tubulin polymerization in juvenile rat brain, placed cellular oxygen consumption by actin and tubulin at 25% and 22%, respectively [42], though we expect that these values are inflated by growth and development. Generally, the contribution of neuronal housekeeping processes in grey matter, including but not limited to cytoskeletal dynamics, is taken to be approximately 25% [43], and this value may be lower for turtle hepatocytes [12]. It follows that the actual values for ATP and GTP consumption by actin and tubulin polymerization are likely much lower than 25%, but in the context of metabolic rate depression, it is arguably more important to look at the big picture of how cytoskeletal dynamics impact cellular ATP consumption. The simple fact that arrest of actin polymerization has the potential to reduce cellular oxygen consumption by upwards of 25% has clear implications for possible strategies of cellular anoxia tolerance.

## 3. The Turtle Cytoskeleton

In those mammals that do not utilize hibernation, brumation, or torpor, the cytoskeletal response to anoxia or low temperature is damaging. Low temperature promotes microtubule depolymerization through the catastrophe reaction [24,45]. In several cell types, hypoxia triggers activation of the Ras homolog family member A (RhoA)/Rho-associated protein kinase (ROCK) pathway, which promotes actin polymerization and remodelling, stress fibre assembly, and actomyosin contraction [46,47]. The formation of contractile actin stress fibres disrupts cell structure and intercellular adhesions [46,48,49,50], and this holds true for hypoxic mouse hepatocytes [49]. The phenotypic response to anoxia is less dramatic for neurons but remains damaging. During acute anoxic or ischemia of pyramidal neurons, the F-actin/G-actin (monomeric, globular actin) ratio increases and promotes aggregation of short F-actin rods in the soma and dendritic trunks [51,52]. These aggregations seemingly disrupt neuronal functioning, as pharmacological inhibition of ROCK activity reduces aggregate formation and increases viability of pyramidal neurons during in vitro ischemia [53]. This suggests that oxygen deprivation over-activates RhoA/ROCK in neurons and that uncontrolled F-actin polymerization contributes to anoxic neuronal damage.

In a cold-tolerant and anoxia-tolerant animal, protection of core microtubule structure for cellular organization and suppression of aberrant RhoA activation would be predicted to minimize tissue damage. Here, we explore the response of the western painted turtle cytoskeleton to anoxic overwintering. The proteome of the painted turtle brain provides support for cytoskeletal adaptations to anoxic overwintering. In anoxic painted turtle brain, β-tubulin and tubulin β-3 chain expression are upregulated, while actin-related protein 3 (involved in F-actin network assembly [54]) expression is downregulated [55]. Additionally downregulated are gelsolin [55], which is involved in calcium-mediated actin dynamics [56], and ALG-2-interacting protein X (Alix; also known as programmed cell death 6-interacting protein) [55], which is associated with F-actin assembly in cell protrusions and focal adhesions [57,58], neurite growth [59], and vesicle fusion and organization [57,60]. Presynaptic neurotransmitter release is inhibited in red-eared turtle brain [61,62], consistent with inhibition of Alix. Inhibition of intracellular vesicle formation and fusion by downregulation of Alix would be expected to contribute to anoxic arrest of protein turnover in red-eared turtle brain [8], and perhaps also in painted turtle hepatocytes [63,64]. Taken together, anoxic protein expression suggests that actin dynamics and scaffolding may be inhibited in anoxic turtle brain. A proteome for 3 °C-acclimated painted turtle heart was recently made available as a pre-print [65], and RhoA was downregulated to 20 °C heart, although this trend was not present in 3 °C-acclimated hatchlings, perhaps due a need for RhoA in development. RhoA protein downregulation in mature overwintering lends further weight to the idea that actin dynamics are arrested during turtle overwintering. The activity of ion transporters, such as Na^+^/K^+^-ATPase [66], are interdependent with the cortical actin cytoskeleton [67,68], raising questions about the regulation of ion channel arrest in turtles. The upregulation of tubulin in anoxic turtle brain is surprising given the energetic cost of microtubule polymerization but may serve to protect cells from calcium-induced loss of microtubule structure or compensate for loss of F-actin structure. Changes in IF abundance were not reported in anoxic turtle brain [55], which may indicate that IFs are not under selective pressure by anoxia, likely because their dynamics do not consume phosphorylated nucleoside triphosphates [13,27] and because studies using anoxia-intolerant mammals indicate that their structure is relatively stable under anoxic [24,69] and cold [24,70] insults. Calcein loading of turtle neurons has revealed that they shrink during anoxia, a phenomenon that is tentatively attributed to chloride and water efflux [71]. We have also observed shrinkage of painted turtle brain sheets upon acute chilling to overwintering temperatures of 4 °C (D. Pyne, unpublished observations) as well as shrinkage of cultured primary turtle hepatocytes incubated either at 4 °C or at 22 °C in cyanide-containing media (A. Myrka, unpublished results; [72,73]). This shrinkage indicates a net depolymerization of cortical F-actin structure, which suggests a possible suppression of RhoA activity. Shrinkage of turtle hepatocytes may contribute to metabolic rate depression, as hepatocyte shrinkage is associated with inhibition of protein synthesis in rats and walking catfish (*Clarias batrachus*) [74,75]. Similarly, chemical anoxia induces an acute 3.2% shrinkage of hepatocytes in another model for anoxia tolerance, the goldfish (*Carassius auratus*) [76]. The anoxic cytoskeleton is otherwise unexplored in goldfish. Anoxic brain volume is maintained in crucian carp (*Carassius carassius*) [77], but loss of memory following anoxia and re-oxygenation implies that an unexplored aspect of anoxia tolerance is neurological repair following overwintering [78]. To understand the potential impact of anoxic cytoskeletal effects of neural interconnectivity, we will contrast anoxia-tolerant models with other overwintering animals.

## 4. Cytoskeletal Shrinkage in Overwintering Animals

While there has been little investigation of the cytoskeleton in anoxia-tolerant animals, research of the neuronal cytoskeleton in cold-tolerant mammals has been relatively abundant, and cytoskeleton-mediated shrinkage of the brain appears to be a widespread adaptation [79,80,81,82,83,84,85,86] that we will explore to inform hypotheses of metabolic arrest in anoxic animals. Mammals that utilize torpor or hibernation depress metabolism while overwintering at low temperatures in burrows that may become hypoxic [87]. Popov et al. [83] first described degradation of dendrites and dendritic spines in torpid Siberian ground squirrels (*Citellus undulatus*) in 1992, but torpid brain shrinkage has since been demonstrated in shrews [80,85] and hamsters [81], and is suspected in black bears [86] as well. During ground squirrel torpor, neuronal protrusions are degraded [83,84]. In the case of Golden-mantled ground squirrels (*Spermophilus lateralis*), torpid neuronal shrinkage is as great as 35–40% by volume [82], accompanied by a 50–65% loss of synapses [88]. This neuronal degradation is reversed upon arousal from torpor, and it is theorized to contribute to metabolic rate depression through reversible degradation of synapses and a consequential reduction of neuronal firing, making its role conceptually similar to that of spike arrest in anoxic turtles (reviewed in [7,89,90]). Synaptic degradation is attributed, in part, to hyperphosphorylation of the microtubule associated protein (MAP) tau, which is hypothesized to cause tau to dissociate from microtubules and destabilize the microtubule structure of neuron protrusions [91,92]. In addition to promoting neurite withdrawal, tau phosphorylation is thought to inhibit N-methyl-D-aspartate (NMDA) receptor activity by a mechanism of cortical F-actin depolymerization [93]. Similarly, tau phosphorylation is argued to promote removal of α-amino-3-hydroxyl-5-methyl-4-isoxazole-propionate (AMPA) receptors from synapses in hibernators (reviewed in [90]). Phosphorylation of tau has been observed in several hibernating mammals [86] and has been described as a “master switch regulating synaptic gain” (reviewed in [91]) in hibernating mammals. In torpid mammals and anoxia-tolerant animals, protein phosphorylation contributes to metabolic rate depression (reviewed in [94,95]). In red-eared turtles, anoxia induces a 60% and 30% increase in 32P labelling of brain and liver, respectively [96], and it is plausible that tau may be among those proteins phosphorylated. Although single cell-level studies of mammalian cytoskeletal shrinkage have been limited to the brain, whole organ-level shrinkage of other organs has been described in shrews. In addition to the brain, the skeleton and, indeed, major internal organs of common shrews (*Sorex Araneus*) also experience reversible shrinkage during torpor (Dehnel’s phenomenon, [97,98]). Organ shrinkage is 34.6% in the case of shrew liver, suggesting that cytoskeletal adaptations to metabolic rate depression may not be exclusive to excitable cells.

While tau phosphorylation destabilizes axonic microtubules [99,100], inhibiting axonal trafficking in the process, protection of some core microtubule organization in the soma would be expected to be critical to cell viability. This is illustrated by the expression of another MAP, microtubule associated protein 2 (MAP2) among overwintering animals. The localization of MAP2 in overwintering animals has been reviewed elsewhere by Gattoni et al. [101], and we have drawn heavily on that review to describe subcellular MAP2 and IF localization here. While neuronal MAP2 expression decreases in the neurons of hibernating ground squirrels [92,102], closer examination reveals that neuronal MAP2 is not simply degraded, but rather MAP2 forms aggregates in the somatic cytoplasm [88]. Such a trend was also observed in dormant edible frogs (*Rana esculenta*) [103]. The evidence of cytoskeletal inhibition in hibernating edible frog neurons is of particular interest to anoxia-tolerant systems, as these animals have freeze tolerance [104] and anoxia-tolerant tissues [105]. The dormant cytoskeleton has also been investigated in the land snail (*Helix aspersa*), an anoxia-tolerant organism [106,107]. As described by Gattoni et al. [101], the same trend seen in dormant frogs is mirrored in dormant snails. Measurement of MAP2 in the neuropile of *Helix aspersa* found that expression was decreased [108], while measurement of the soma of the garden snail (*Cornu aspersum*) in another study found that expression was increased [109]. Gattoni et al. [101] suggested that this pattern in frogs and snails supports re-localization of MAP2 from cell neurites to the soma. Such a pattern of MAP2 expression suggests increased microtubule stability in the soma relative to neurites, and this pattern is mirrored by subcellular distribution of tau phosphorylation.

In hibernating Syrian hamsters (*Mesocricetus auratus*), pyramidal neurons tau phosphorylation is more prevalent in distal regions of apical dendrites than in basal dendrites or in the soma [110,111]. This suggests not only relatively high microtubule stability in the soma, but also that overwintering microtubule stability differs among dendrite subpopulations. The preservation of tau binding to microtubules in basal dendrites may be indictive that inhibitory synapses are maintained. In the basal forebrain of hibernating Syrian hamsters, cholinergic neurons express phosphorylated tau, while tau phosphorylation is largely absent in γ-amino butyric acid (GABA)ergic neurons [112]. In painted turtles [44,71] and goldfish [113], GABAergic inhibition of pyramidal cells by less abundant stellate cells [114,115] is a key mechanism of anoxia tolerance. If selective phosphorylation of tau occurs in anoxic turtles and fish, as it does in hamsters, then excitatory synapses could be inhibited while relatively less abundant inhibitory synapses are maintained. This predicts two levels of control over anoxic synaptic gain, where selective regulation of synapse morphology complements and contributes to established mechanisms of spike arrest through selective regulation of neurotransmitter activity [7].

The most abundant IFs in neurons are neurofilaments (NF; [116]), which crosslink with microtubules [117,118] and provide most of the cytoskeletal volume in developed neurons [116]. Studies of NF in overwintering animals have been reviewed by Gattoni et al. [101] and will be briefly summarized here. Phosphorylation of the NF heavy chain (pNFH) promotes localization to axons, resulting in axon stability and assembly of axon cytoskeleton [117,118,119,120]. Overwintering dormancy promotes increased NF protein abundance in ground squirrels [79], but decreased pNFH abundance was demonstrated in hedgehogs and frogs [101,103,121]. Ground squirrel hypothalamus proteomics also showed an increase in cytokeratins during hibernation, the implication of which is less clear [102]. As with MAP2, subcellular pNFH abundance during dormancy in snails [108,109] suggested a re-localization of IF from neurites to somatic stores during dormancy. Decreased pNFH abundance predicts a reduction of neurite volume, while increased total IF may contribute to somatic rigidity and a compensatory protection of organization in the soma, much like what we have suggested for microtubules in anoxic turtle brain.

Tau and MAP2 also facilitate F-actin bundling and cross-linking, microtubule and F-actin crosstalk, neurite growth [122,123,124,125,126,127,128,129], and reduction of these MAPs and pNFHs at the cell periphery predicts inhibition of peripheral F-actin structure, which may facilitate cell shrinkage and a more globular and microtubule-dominated phenotype. We argue that the above patterns facilitate arrest of protrusions and synaptic contacts while maintaining core somatic intracellular structure, and that these adaptations may be present in anoxic turtles. Such a strategy could contribute to the morphological synaptic arrest observed in hibernators as well as spike arrest in anoxia-tolerant animals but be reversible by virtue of protecting the soma. This strategy could also explain why, despite a theorized reduction in neurite microtubule structure, anoxic turtle neurons have an increased total β-tubulin protein abundance [55]. Similarly, increased α-tubulin protein content was reported in hibernating 13-lined ground squirrel (*Ictidomys tridecemlineatus*) neurons [79]. Another investigator found a decrease in hibernating α-tubulin protein abundance of little ground squirrel (*Citellus pygmaeus*) neurons [102], but this study was conducted on the hypothalamus, while the former examined the forebrain, suggesting that the response of microtubule abundance varies by brain region. While the above research has been limited to neurons, the logic behind this strategy of metabolic rate depression could easily be applied to other tissues and is hinted at by the shrinkage of multiple major organs in shrews, including liver [94]. Actin dynamics participate in cell cycle progression/tissue growth [130] and general aspects of cellular energy expenditure [35,131]. In hepatocytes, actin dynamics enable such metabolically active processes as bile, albumin, cholesterol secretion [132,133,134], and canicular contraction [135]. Thus, primary turtle hepatocytes provide a model for testing overwintering tau phosphorylation and cytoskeletal arrest in non-excitable cells.

## 5. Calcium Signalling Is Associated with Cytoskeletal Inhibition

Modifications to the neuronal cytoskeleton of hibernators are associated with calcium binding proteins in both vertebrates and invertebrates (reviewed in [101]). In 2018, Gattoni et al. [109] found that aggregates in the neuronal cytoplasm of dormant garden snails display colocalized immunoreactivity for phosphorylated tau and the calcium binding protein calmodulin (CaM), and proposed a theory as follows: CaM-dependent kinases phosphorylate tau in a calcium-dependent manner [99,136,137]; further, CaM can bind to tau directly [138,139], inhibiting binding of tau to microtubules [140]. Dormant neurons also contained aggregates with MAP2 immunoreactivity, as was mentioned above, which appeared qualitatively similar to the CaM aggregates, but colocalization of these immunoreactivities was not tested [109]. Similar to tau, calcium-activated CaM binds to MAP2, triggering dissociation from microtubules and reduced microtubule stability [139,140]. These results led the authors to propose that tau-mediated microtubule depolymerization in dormant snail neurons might be regulated by calcium activation of CaM, and that the same might be true for MAP2-mediated depolymerization [109]. CaM also facilitates NF phosphorylation [141] and colocalized with NFs in garden snails [109]. Together with the findings that CaM and pNFH levels are correlated in dormant edible frogs [103] and garden snails [108], this suggests a role of calcium in regulating NF organization in overwintering, anoxia-tolerant animals. This was the first study to examine tau regulation in non-mammalian overwintering and raised the possibility that cytoskeletal inhibition mediated by MAPs is a widespread adaptation to metabolic rate depression, at least in neurons, begging the question as to whether such adaptations extend to anoxia tolerance among animals.

## 6. Cytosolic Calcium Increases Marginally in Anoxic Turtle Cells

In anoxia-intolerant cells, oxygen depletion results in a pathological elevation in cytosolic calcium due to release from intracellular stores, including the endoplasmic reticulum and mitochondria [142,143], or from increased cellular uptake [144,145,146], depending on cell type. Similarly, hypothermia triggers cellular calcium overload, at least some of which is due to uptake of extracellular calcium, but likely involving release of intracellular calcium stores as well [147,148,149,150]. Calcium overload leads to cytotoxic mitochondrial damage [151,152] as well as degradation and aberrant contraction of the cytoskeletal elements [153,154]. Anoxia-tolerant animals, including turtles, avoid calcium overload [155,156], but a smaller, vestigial rise in calcium may serve a newfound role in the signalling of metabolic rate depression, similar to that described in overwintering animals above. We next summarize the role of calcium in metabolic rate depression of anoxic turtle cells.

Bickler and Buck [155] argued that NMDA receptor inhibition in anoxic western painted turtle neurons was calcium dependent, likely acting on F-actin stability, and a similar mechanism is likely present for AMPA receptors [7]. High intracellular calcium can trigger F-actin depolymerization through the actions of a variety of proteins, such as gelsolin [56,157], and such depolymerization inhibits NMDA receptors [32,158]. Alternatively, the calcium may act upon α-actinin to separate ion channels from intact cortical F-actin [159,160]. It follows that calcium-dependent modulation of F-actin stability may contribute to anoxia tolerance through ion channel arrest. Mildly elevated cytosolic calcium is a property of not only anoxic turtle neurons [161], but generally of hibernating animal neurons at low temperature, as we have discussed [101,162]. It is therefore important to consider the regulation and origin of this calcium in anoxia-tolerant systems.

In 1992, no cytosolic calcium change was detected by Bickler in turtle neurons within 30 min of anoxia using fura-2 [163]. Bickler and Buck [164] did not observe an anoxia-mediated acute increase of calcium in turtle neurons, but they did report a wide variation in baseline calcium measurements. Fura-2 imaging after longer exposure later showed that cytosolic calcium did increase by 35% after 2 h of anoxia [159], and that calcium increase was maintained over 40 days of anoxia [reviewed in 155]. Concurrent with calcium increase, CaM activity inhibited NMDA receptor activity [159]. Adenosine reduces NMDA receptor activity in turtle neurons [165] and decreases oxygen consumption in turtle hepatocytes (R. Centritto, and L.T. Buck, unpublished results; [166]). Further, adenosine inhibits Na^+^/K^+^-ATPase activity in goldfish hepatocytes, suggesting ion channel arrest following adenosine exposure [167]. Bickler and Buck [10] therefore proposed that the source of anoxic calcium might be adenosine-stimulated activation of the inositol 1,4,5-trisphosphate (IP3) pathway and consequential calcium release from the endoplasmic reticulum (ER) [10]. An alternative explanation was that the calcium originated in the mitochondria [161].

**Figure 2:**
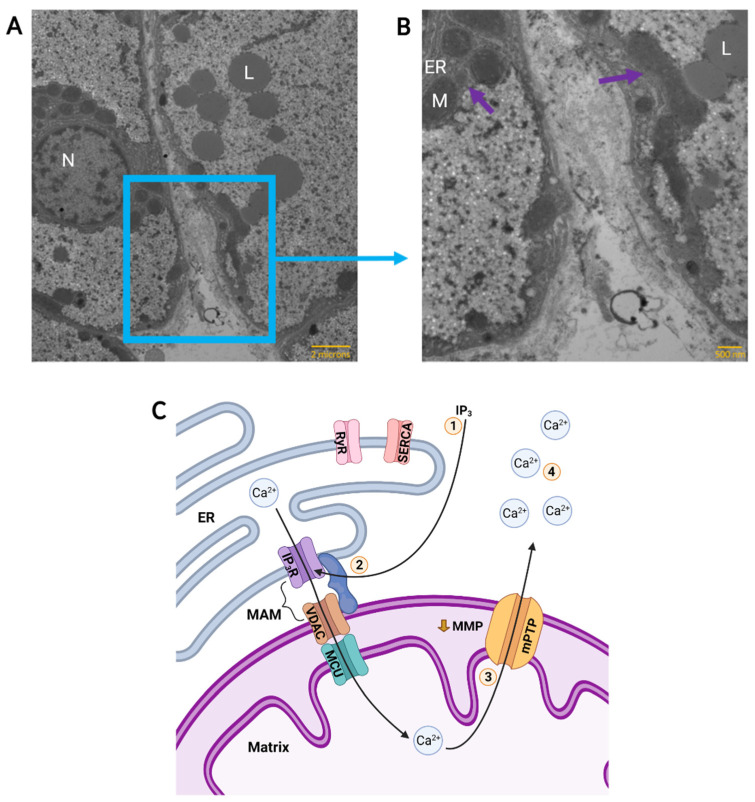
Calcium Flux Through Mitochondria-Associated Membranes (MAM) in an Anoxic Turtle Cell. A representative Transmission Electron Micrograph of two adjacent normoxic hepatocytes in a turtle liver slice is shown in (**A**) and the boundary between two cells is enlarged in (**B**). Structures of interest are indicated; N: nucleus, L: lipid droplet, ER: endoplasmic reticulum, M: mitochondrion. Likely sites of MAM, as evidenced by endoplasmic reticulum and mitochondria in close proximity, are indicated (purple arrows). Scale bars are 2 microns (**A**) and 500 nanometres (**B**). Anoxic calcium flux is depicted in (**C**). **1**: Following adenosine signalling, the inositol 1,4,5-trisphosphate (IP_3_) pathway activates ER IP3 receptors (IP_3_R) in the MAM complex. Ryanodine receptors (RyR) and Sarcoendoplasmic Ca^2+^-ATPase (SERCA) are not activated. **2**: ER IP_3_R and mitochondrial voltage-dependent anion channels (VDAC) may be connected by MAM, permitting transport of Ca^2+^ from the ER and into the mitochondrial matrix through mitochondrial calcium uniporter (MCU). **3**: Anoxic partial depolarization of mitochondrial membrane potential (MMP) triggers formation of a low conductance form of the mitochondrial permeability transition pore (mPTP). **4**: Ca^2+^ is released into the cytosol causing a mild elevation of cytosolic calcium. (**A**,**B**) modified with permission from images obtained by D. Hogg, unpublished results. Samples were isolated as part of another study that was approved by the University of Toronto Animal Care Committee, and turtles were supplied by Niles Biological (Sacramento, CA, USA) and housed as described previously [44]. (**C**) created with BioRender.com.

Pamenter et al. [168], using fura-2 acetoxymethyl (AM) ester, obtained results indicating an anoxic calcium increase of 9.3%. They then used inhibitors of ER calcium release through ryanodine receptors (RyR) and Sarcoendoplasmic Ca^2+^-ATPase (SERCA). They found no effect with these inhibitors, and no change in the calcium response when using calcium-free media, and therefore concluded the calcium to be mitochondrial in origin. Using Oregon green-1,2-bis(2-aminophenoxy)ethane-N,N,N′,N′-tetraacetic acid (BAPTA), a more sensitive calcium indicator than fura-2 [169], Hawrysh and Buck [161] demonstrated again that acute anoxia on the order of minutes does increase cytosolic calcium in painted turtle neurons and presented evidence that mitochondrial calcium release occurred through a low conductance form of the mitochondrial permeability transition pore (mPTP) following ATP-sensitive potassium influx and partial depolarization of mitochondrial membrane potential (MMP). This theory is not entirely incompatible with that of adenosine triggered, IP_3_-mediated calcium release; both theories are compatible if calcium is shuttled from the ER to the mitochondria through the mitochondria-associated membranes (MAM; [170]). When the ER and mitochondria are in close proximity, the MAM complex can form between ER IP_3_ receptors (IP_3_R), mitochondrial voltage-dependent anion channels (VDAC), and several associated proteins [170]. This provides a possible consolidation of previous results, wherein the mitochondria act as a “gatekeeper” for the release of stored calcium into the cytoplasm (Figure 2C). The oresence of this structure in turtles has not been investigated, but Figure 2A,B (D. Hogg, unpublished results) shows that the ER and mitochondria in painted turtle hepatocytes are in very close proximity, as would be expected if MAM is found in these cells.

## 7. Reduced ROS Production in Anoxic Animals May Protect Microtubules from Catastrophic Loss of Structure

We have described above how cold or anoxia-induced cytoskeletal damage in anoxia-intolerant animals is attributed, in part, to calcium overload, and reactive oxygen species (ROS) production is an integral part of the problem. In anoxia-intolerant, non-hibernating animals, both the transition to anoxia [48,171,172] and reduced temperature [173,174,175] are associated with increased ROS production, and reoxygenation further increases ROS as accumulated succinate stores are oxidized [176]. The problem is compounded by a reduction of ROS scavenging due to depletion of antioxidants and failure of antioxidant translation [172,174,177]. An overload of intracellular calcium and elevated ROS promote each other in a positive feedback loop. High ROS concentration mediates ER IP_3_ and RyR-mediated calcium release into the cytosol. Subsequent uptake of calcium by the mitochondria promotes further mitochondrial ROS production, which increases ER stress. ROS can also trigger the transfer of calcium from the ER into the mitochondria directly through the MAM [178]. It follows that sources of both calcium and ROS must be mitigated in order to avoid calcium overload and associated aberrations of the cytoskeleton during anoxia. In addition to promoting calcium-mediated cytoskeletal degradation, high ROS levels also reduce cytoskeletal integrity directly (reviewed in [179]). Like high cytosolic calcium [180], high cytosolic ROS causes depolymerization of microtubules [179], severing of F-actin [179], as well as degradation of IFs [181], and it results in the collapse of axonal growth cones [179]. Overwintering animals must therefore depress ROS formation and these mechanisms will be considered next.

Using induced pluripotent stem cells (iPSCs) isolated from 13-lined ground squirrels and reprogrammed into neurons, Ou et al. [182] showed that the mitochondria of squirrel-derived cells partially depolarized with chilling to 4 °C and cellular protein oxidation did not increase. The same investigators showed that upon chilling to 4 °C, human IPSC-derived neurons experienced mild mitochondrial hyperpolarization and microtubule depolymerization. They were able to prevent these effects using a mitochondrial uncoupler and protease inhibitors. The authors then showed that this treatment was sufficient to prevent microtubule depolymerization in mouse kidneys stored at 4 °C for 24 h. Finally, they concluded that during chilling in the human neuron model, mitochondrial hyperpolarization triggers excess ROS production, which both damaged microtubules directly, and damaged them indirectly through ROS-induced rupture of lysosomes. The results suggested that ground squirrel cells have the capacity to depress ROS production by partially depolarizing their mitochondria at low temperature, and that this protection is sufficient to protect microtubules from cold-induced depolymerization [182]. This is consistent with results from torpid 13-lined ground squirrel muscle and liver, in which ROS generation is suppressed [183]. Similarly, the mitochondria of cultured Syrian hamster kidney (HaK) cells maintain membrane potential at 10 °C and avoid an increase in ROS production [184].

Not all human cell types have mitochondria that hyperpolarize with acute chilling. The mitochondria of chilled human embryonic kidney 293 (HEK293) cells depolarize and still experience an increase in oxidation [184]. Although hyperpolarization increases ROS production by promoting oxidative phosphorylation, depolarization can increase ROS as well [185], especially at Complex 1 and Complex 3 [186]. With severe oxygen deprivation, anoxia-intolerant mitochondria severely depolarize [187,188], and ROS production is increased [48,171,172]. In summary it appears that excess ROS production, likely following a failure of MMP regulation, may be a key element of cytoskeletal damage caused by cold and anoxia in intolerant systems. We will next contrast these effects with ROS levels reported in anoxia-tolerant animals.

Although anoxic painted turtle neuronal mitochondria depolarize [10], this depolarization does not result in increased ROS production; rather, ROS production decreases by 25% [189], likely following mitochondrial calcium release [190]. This reduction contributes to neuronal anoxia tolerance, as indicated by cell viability [191]. ROS scavenging of painted turtle neurons mimics anoxic signalling by facilitating inhibitory GABAergic signalling [44,192]. Similarly, ROS production decreases by 10% with the onset of anoxia in goldfish brain, and ROS scavenging mimics anoxic inhibition of excitatory actional potential firing [193]. In the heart of cold-acclimated and anoxic red-eared turtles, ROS formation from succinate is suppressed by maintenance of a low succinate to fumarate ratio and preservation of ATP pools through metabolic suppression [194]. Supercomplex formation of electron transport chain (ETC) complex one and other ETC complexes can reduce both ROS generation and oxygen consumption in neurons [195], although no change in supercomplex composition was detected in anoxic or 4 °C red-eared turtle heart [196]. In addition to depression of ROS generation, anoxic red-eared turtles have high constitutive activities of antioxidant defences relative to similarly sized ectotherms, and activities are on par with those of similarly sized mammals [197,198,199].

Reduction of ROS formation by overwintering animals likely goes a step beyond just avoiding ROS-induced cytoskeletal damage. Depleted ROS levels inhibit F-actin dynamics and decrease neurite outgrowth in a manner consistent with demonstrated neurite retraction in hibernators [82,88,179,200]. Low cellular ROS is yet another mechanism by which F-actin content and dynamics can be inhibited [reviewed in 179]. Depletion of ROS below normal levels results in reduced formation of filipodia, lamellipodia, and axonal growth cones in developing neurons [179,201]. Relatively high, non-pathological ROS abundance is associated with actin-mediated cytoskeletal remodelling [reviewed in 202], and suppression of ROS generation may inhibit these ATP-demanding activities. The impact of ROS depression on microtubule dynamics has not yet been characterized [202]. RhoA function is impacted by oxidation, with oxidation by high ROS increasing activity, but the impact of decreased ROS on RhoA is yet unknown [202]. With the exception of severe mitochondrial depolarization, ROS production is correlated with magnitude of the MMP [186,203,204], and partial depolarization of mitochondria, as is the case in anoxic turtles [189] and 4 °C ground squirrels [182], may serve to limit ROS production.

## 8. Gasotransmitters Involved in Anoxic Metabolic Rate Depression and Their Impact on Cytoskeletal Dynamics

The membrane-permeable [205] gasotransmitters hydrogen sulphide (H_2_S), nitric oxide (NO), and carbon monoxide (CO) are of increasing interest in anoxia tolerance (reviewed in [206]), and all three have documented regulatory effects on MMP, ROS production, and cytoskeletal dynamics in mammals. Therefore, their functions, as well as their levels in overwintering animals and anoxic turtles, warrant consideration. H_2_S is produced by cytosolic cystathionine γ-lyase (CSE) and cystathionine β-synthase (CBS), as well as mitochondrial 3-mercaptopyruvate sulphur-transferase (3-MST). NO is produced by nitric oxide synthases (NOSs) and CO is produced by heme oxygenases (HOs). Most NOS is cytosolic, while most CO is present in the ER, but activity of these enzymes is found in the mitochondria as well [207]. Heme oxygenase 2 (HO2) has been detected in the mitochondria of mouse hepatocytes and is associated with hypoxia [208]. NOS activity is documented in mitochondria, although the isoform identity of this mitochondrial NOS is a topic of debate [209]. Similarly, in response to hypoxia, CBS accumulates in mitochondria [210]. All three gasotransmitters inhibit oxidative phosphorylation at complex four [211] and, depending on concentration, CO can also inhibit complex one and NO can inhibit all five complexes of the ETC (reviewed in [212]). HO1 is upregulated in multiple tissues of hibernating 13-lined ground squirrels, including brain and liver [213]. Recently it was determined that H_2_S availability is increased in hibernating 13-lined ground squirrel liver, contributing to overwintering suppression of the ETC [214]. In addition to mitochondrial ETC inhibition, gasotransmitters can open mitochondrial potassium channels [215], which promotes depression of MMP and the reduction of ROS production, as will be discussed below. Further, all three gasotransmitters can scavenge ROS either directly, or indirectly, via activation of ROS scavenging pathways [207].

Gasotransmitter function is most commonly known by the relaxation effect it has on smooth muscle, a phenomenon of actomyosin relaxation that occurs through a variety of pathways (reviewed in [205]). Indeed, the three gasotansmitters all have inhibitory effects upon RhoA, a protein which, as was described above, promotes actin dynamics and cellular actomyosin contraction. Effects of gasotransmitters are induced, in part, through post-translational modifications: S-sulfhydration by H_2_S, S-nitrosylation by NO, and carbonylation by CO [216]. H_2_S inhibits RhoA actomyosin contraction [217,218], seemingly through S-sulfhydration [219]. In neurons, H_2_S can inhibit RhoA through an unknown mechanism of RhoA phosphorylation, and this modification confers increased resistance to hypoxic/reoxygenation injury [220]. S-nitrosylation of RhoA by NO inhibits its activity, reducing actin stress fibre assembly [221,222] and myosin contraction [223] by preventing GTP binding to RhoA [223,224]. In the human fibroblast model this inhibition is sufficient to prevent formation of lamellipodia and filipodia [225]. CO inhibits RhoA by preventing GTP binding [226], perhaps by carbonylation, and suppresses actin dynamics and formation of cell protrusions [227,228]. NO causes axon retraction and inhibits the kinesins kinesin-related protein 5 (KIF5) and kinesin family member 21B (KI21B). It is hypothesized that NO inhibition of kinesins inhibits vesicle trafficking necessary for axon growth [200].

Only H_2_S and NO have been investigated in turtles (reviewed in [206]). Free H_2_S decreases with cold acclimation in red-eared turtle liver and bound H_2_S increases with cold acclimation in turtle brain. Strangely, the combination of cold and anoxic acclimation results in free and bound H_2_S levels that do not differ significantly from normoxia. In turtle erythrocytes cold increases both free and bound H_2_S, but there is no further increase with cold anoxia [229]. This suggests a possible role of circulating H_2_S in cold-tolerance, but implications for anoxia are unclear. In both the red-eared turtle and the anoxia-tolerant crucian carp, tissue-specific expression of CSE, CBS, and 3-MST is maintained during anoxia or cold acclimation [230], but enzyme activities were not reported. Whole-tissue measurements of metabolites are not able to capture every nuance of intracellular signalling and chronic and acute exposure to anoxia may have differing effects. In painted turtle brain sheets, inhibition of CSE, CBS, or 3-MST reduces the anoxia-mediated cytosolic calcium increase [231]. Normoxic administration of exogenous H_2_S stimulates an increase in cytosolic calcium and endogenous H_2_S concentration increases during anoxic exposure [231]. This suggests a role for H_2_S production in anoxic calcium signalling in turtle brain. Coupled with the above finding of increased bound H_2_S in cold-acclimated turtle brain [229], H_2_S is implicated in the regulation of metabolic depression in turtles. Whether H_2_S-induced calcium increase disrupts the cytoskeleton, or whether H_2_S inhibits RhoA, remains to be tested in turtles.

Circulating NO metabolites increase in red-eared turtle circulation during anoxia [232]. Similarly, nitrite increases in anoxic red-eared turtle heart, muscle, and erythrocytes and nitrite metabolites increase as well, suggesting a role of NO in anoxia tolerance in turtles [232]. In painted turtle brain sheets, acute anoxia causes a decrease in NO production, which was mimicked by blocking NMDA receptor activity, suggesting that NO production is downregulated following ion channel arrest in the turtle brain [233]. This result mirrors suppression of inducible NOS in hibernating Arctic ground squirrel brain forebrain [234]. Nitrite and NO metabolites are upregulated by anoxia in the heart of crucian carp, with some NO metabolites upregulated in other tissues as well [235]. Lastly, nitrite and nitrate are upregulated in tissues of hypoxic or dormant anoxia-tolerant wood frogs [236].

HO1 is upregulated by anoxia in red-eared turtle brain [237]. In goldfish, HO1 activity, but not protein expression, is upregulated in gill by hypoxia in fish acclimated to 7 °C, but not in hypoxic fish acclimated to 25 °C [238]. This suggests that CO signalling is involved in the goldfish response to the combined challenges of cold and hypoxia.

**Figure 3:**
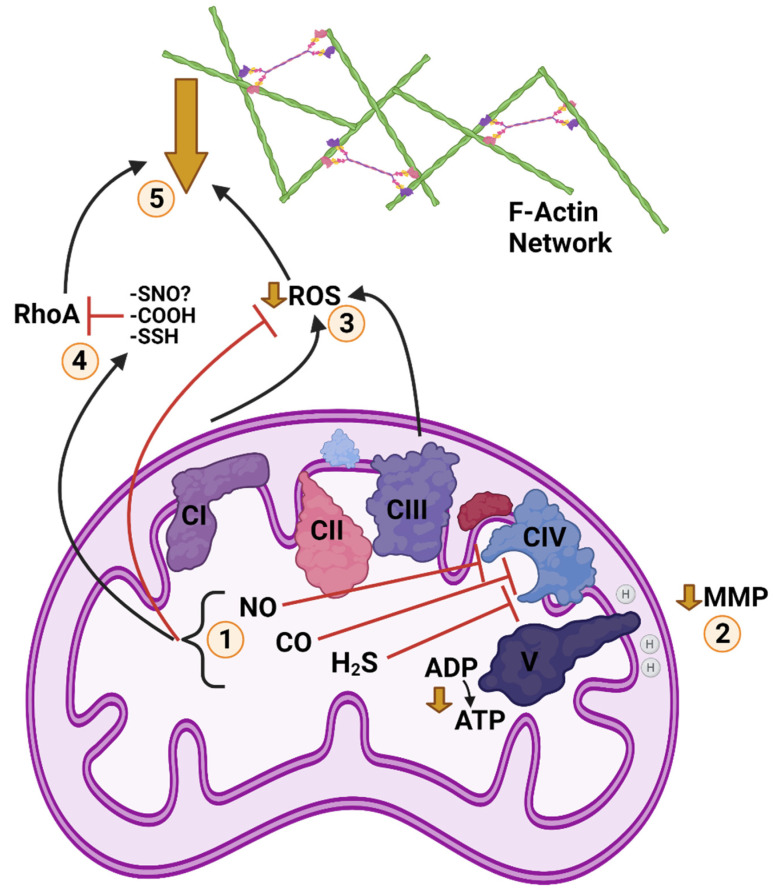
Posited Cold Anoxic Regulation of F-Actin by Gasotransmitters and Reactive Oxygen Species (ROS) in a Turtle Cell. **1**: Cold and anoxia modulate abundance of nitric oxide (NO), carbon monoxide (CO), and hydrogen sulphide (H_2_S), which inhibit mitochondrial complex four. **2**: Inhibition of the electron transport chain (ETC) at CIV reduces ATP synthase activity, resulting in a partial depolarization of mitochondrial membrane potential (MMP). **3**: Reduction of ETC activity/MMP also reduces ROS generation. Scavenging by the action of gasotransmitters further contributes to reduced ROS. **4**: NO, CO, and H_2_S post-translationally modify Ras homolog family member A (RhoA) by S-nitrosylation, S-sulfhydration, and possibly carbonylation (mechanism of CO inhibition unconfirmed), respectively, inhibiting RhoA activity. H_2_S can also inhibit RhoA by triggering RhoA phosphorylation. **5**: Inhibition of RhoA reduces stability of F-actin networks and inhibits structural rearrangement and actomyosin contraction. Reduction of ROS signalling decreases F-actin abundance and assembly. Together, these changes inhibit F-actin dynamics and structural rearrangement during anoxia. Created with BioRender.com.

Our current understanding of gasotransmitter abundance in anoxia-tolerant animals is not clear-cut, as available evidence is limited. It can be concluded that metabolism surrounding these intermediates does respond to anoxia in turtles and fish, and H_2_S and NO production respond to anoxia in turtle brain, but the tissue-specific effects and their downstream consequences lack clarity at this time. The finding that H_2_S is involved in anoxic calcium abundance in painted turtle brain is intriguing, as it potentially implicates H_2_S in mitochondrial oxygen sensing and ROS regulation. Each of the gasotransmitters can inhibit oxidative phosphorylation, and therefore could theoretically contribute to mitochondrial depolarization and calcium release, as well as suppression of ROS generation. As gasotransmitters have the capacity for RhoA inhibition, and we predict actin dynamics to be downregulated in anoxic turtles as described above, mitochondria are shaping up as a signalling hub for anoxic cytoskeletal regulation. Potential anoxic interactions of gasotransmitters, ROS, MMP, and actin dynamics in turtles are summarized in Figure 3.

## 9. MMP Depolarizes Marginally in Anoxic Turtle Cells

As detailed above, mitochondrial calcium release in anoxic painted turtle neurons occurs through the mPTP following a partial depolarization of mitochondrial membrane potential [161]. As mitochondrial ATP supply falls, mitochondrial ATP-sensitive potassium channels are activated, resulting in mitochondrial potassium uptake [168], depolarizing MMP by 10–20% [168], reducing MMP-driven mitochondrial calcium uptake [168], and triggering MMP-sensitive formation of the mPTP [161]. Partial depolarization of the MMP has also been demonstrated in painted turtle hepatocytes using cyanide and rhodamine, but no corresponding increase in calcium was observed using Oregon green-BAPTA [239], nor was a change in calcium seen with oxygen tensions down to 0.1 Torr (E. Lari, unpublished results). It is possible that hepatocyte calcium release occurs after a longer anoxic duration or with cold exposure, but these are avenues for future research. If MMP in turtle cells were to depolarize completely in the absence of oxygen-dependent proton pumping, the organelle would not be able to maintain ion homeostasis, triggering cytochrome C release and pro-apoptotic signalling [240]; therefore, an alternate source of proton pumping must be mobilized in order to maintain anoxic MMP. In turtles, anoxic proton pumping is widely regarded to occur through reversal of ATP synthase and at the expense of ATP [161,194,239,241,242,243,244], though this has not yet been demonstrated in non-excitable turtle cells such as hepatocytes. This reversal makes the mitochondria an energetic “liability” during anoxia when ATP supply is limiting [245].

## 10. Mitochondrial Distribution and Dynamics on the Anoxic Cytoskeleton

How mitochondrial distribution and dynamics are impacted by cytoskeletal changes during anoxia, or by the demand of anoxic mitochondria for ATP, are open questions that we will consider. Hypoxia in anoxia-intolerant mammals is characterized by hypoxia-inducible factor (HIF)-dependent perinuclear localization of mitochondria facilitating nuclear ROS accumulation [246,247]. Increased ROS formation does not occur during an anoxic transition in painted turtles [189], so perinuclear localization for this purpose would not be expected. Depolymerization of peripheral microtubules, such as may occur in retracting axons, would predict fission [248] and an inward movement of axonic mitochondria. Axonic mitochondria and vesicles are trafficked along microtubules [249,250]. Retraction of neurites and cytoskeletal structure may arrest trafficking activity, as has been suggested of dormant garden snail neurons [108]. Motor proteins are powered by ATPase activity, and how a reduction of peripheral structure, and therefore trafficking, might impact cellular ATP consumption is unexamined. Interestingly, the proteome of mature 10 °C-acclimated painted turtle heart has decreased protein abundance of dynactin subunit 2 [65]. Dynactin is required for the functional attachment of dynein to cargo and also contributes to kinesin trafficking activity [251]. The depression of this protein with cold acclimation hints at regulation of intracellular trafficking; however, this depression was not present in cold-acclimated hatching turtles [65]. A reduction in trafficking of cellular cargo would be consistent with the reduction in protein turnover seen in anoxic turtles, as evidenced by reduced protein degradation and protein synthesis [8,63,64].

In anoxia-intolerant mammals, hypoxia induces mitochondrial fission and mitophagy [252,253], and these processes may be active during anoxia in turtles in order to reduce ATP demand for mitochondrial maintenance by reversal of ATP synthase [161,194,239,241,242,243,244]. In cold- and anoxia-intolerant mammals, MMP depolarization inhibits mitochondrial fusion [253,254,255] and thus favours fission. Decreased mitochondrial interconnectivity is associated with higher resistance to ROS injury [253,256], as well as decreased mitochondrial ROS production, likely by virtue of decreased oxygen consumption [257]. The degree of mitochondrial interconnectivity is thought to be a means of regulating oxidative capacity and accompanying ROS production, with mitochondrial networks forming and fragmented in response to the cell cycle [258] as well as cellular circadian rhythm [257]. In contrast, when a fragmented mitochondrial phenotype is accompanied by very high or very low MMP, particularly during a disease state, high ROS production occurs, and this is the case in anoxia-intolerant mammals exposed to damaging hypoxic [259].

An anoxia-tolerant animal does not benefit from oxidative capacity during an anoxic period and might therefore reduce mitochondrial interconnectivity as protection against ROS formation during reoxygenation. *C. elegans* possesses an anoxic stress response whereby when it enters suspended animation mitochondrial fragmentation through fission is upregulated, and mitochondrial size and number decrease in a HIF-1-independent manner. Mitochondria later recover by fusion upon reoxygenation. The response in *C. elegans* is thought to be activated by oxidative stress during the anoxic transition and to be dependent upon AMPK activation [260]. Mitochondrial fragmentation is promoted by AMPK [261], which is under tissue-dependent anoxic regulation in red-eared turtles [262] and crucian carp [263]. Similarly, the liver mitochondria of hibernating long-tailed ground squirrels (*Citellus undulatus*) take on a shrunken, condensed state relative to non-hibernating animals [264] and are thought to increase respiration by swelling in the Spring [265]. In red-eared turtle heart, a large decease in anoxic mitochondrial protein synthesis, relative to a lesser reduction of whole-tissue protein synthesis, suggests an arrest of mitochondrial growth [266]. On the other hand, the presence of F-actin is required for mitochondrial fission, so a reduction in actin dynamics might predict less fission [19]. The filaments involved in cytosolic mitochondrial fission [19] are, however, fewer and discrete from the cortical F-actin cytoskeleton described above and, as with all theories of metabolic arrest, we would predict a reduction in activity, not the complete absence of it.

Another potential level of control is found at intermitochondrial junctions, which are suggested to increase bioenergetic efficiency [267] and have been detected among animals ranging from mammals to molluscs [267]. Bundgaard et al. [196] examined electron micrographs of 2D sections of red-eared turtle hearts and found that cold and anoxia did not decrease mitochondrial volume or cristae surface area; however, their representative images appeared to show reduced intermitochondrial contact in samples from anoxic and cold/anoxic turtles. As intermitochondrial junctions were not the subject of the study, this may simply be sampling bias of the images, but anoxic mitochondrial interconnectivity and junctions present an avenue for future investigation. Bundgaard et al. [196] found no change in the abundance of ETC complexes one, two or five in cold or anoxic turtle heart [196]; however, Farhat et al. [268] found a decrease in cytochrome c oxidase respiration in all tissues tested in anoxic goldfish, except for heart. They concluded that the decrease in complex four respiration was likely indicative of a decrease in mitochondrial abundance. Future imaging studies will be needed to test whether complex four respiration is indicative of mitochondrial density in goldfish; Bundgaard et al. [196] found that anoxia decreased turtle heart complex one activity without a concomitant decrease in protein density [196].

A similar study by Hendriks et al. [184], cited above in the context of ROS, was performed in a hibernator, comparing the effects of cold temperature on the mitochondria of cultured HaK (hamster kidney) cells and HEK293 cells. In HEK293 cells, chilling resulted in mitochondrial fission and decreased interconnectivity. In HaK cells, chilling did not induce fission, but mitochondrial interconnectivity in all treatments was relatively low and like that of chilled HEK293 cells. The authors proposed that constitutively low interconnectivity in hibernator cells might be protective by facilitating rapid turnover of damaged mitochondria by lysosomal mitophagy, a process that requires discreet mitochondrial particles and is facilitated by fission [269]. They also alluded to the possibility of constitutively low oxidative capacity being protective. If a constitutively fragmented mitochondrial phenotype, which minimizes ROS production [257], were present in anoxia-tolerant animals, it would complement constitutively high ROS scavenging capacity [197,198,199]. The contribution of mitochondrial abundance and phenotype to metabolic rate depression in turtle cells, and how such regulation is organized in the context of hypothesized changes in overwintering and anoxic cytoskeletal architecture, is an area deserving of greater attention.

## 11. Summary of Cytoskeletal Arrest Theory

**Figure 4:**
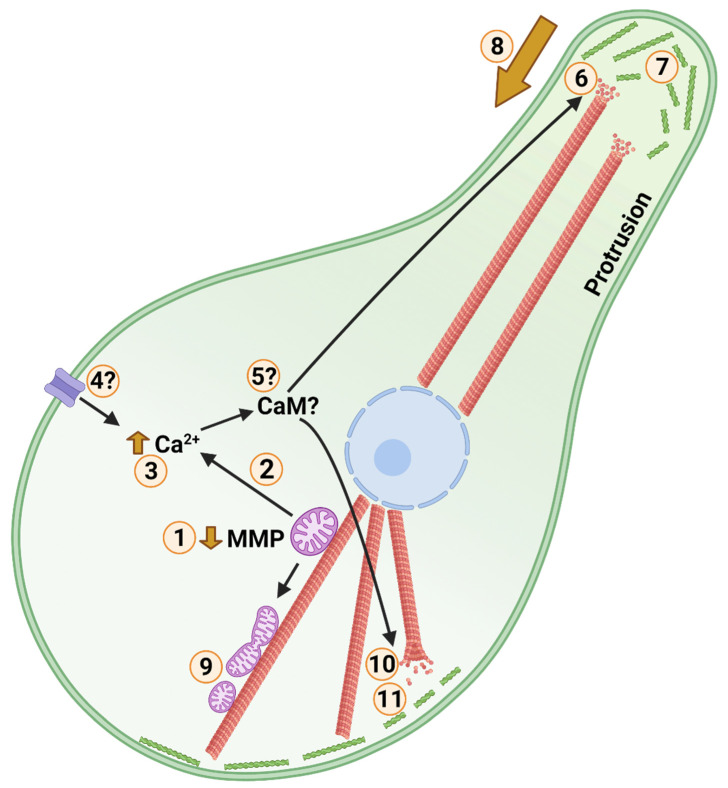
Posited Events of Cold Anoxic Cytoskeletal Arrest in a Turtle Cell. Cortical F-actin, microtubules, mitochondria, and the nucleus are shown in green, red, purple, and blue, respectively. Low temperature and anoxia act as environmental stimuli. **1**: Following environmental stimuli, mitochondrial membrane potential (MMP) is partially depolarized. During anoxia this is accomplished by activation of ATP-sensitive potassium channels. **2**: Calcium is released through a low conductance form of the mitochondrial permeability transition pore. **3**: Cytosolic calcium level is increased marginally. **4**: Calcium influx may occur through membrane calcium channels in response to cold. **5**: Unknown calcium signalling influences the cytoskeleton, potentially involving calmodulin (CaM), gelsolin, microtubule associated proteins (MAPs), and Ras homolog family member A (RhoA). **6**: Plus ends of peripheral microtubules, such as those in neuronal axons, are destabilized. **7**: Peripheral F-actin stability and dynamics are inhibited. **8**: 6 and 7 together facilitate protrusion withdrawal and shrinkage of cell area. **9**: Partial depolarization of mitochondria inhibits mitochondrial fusion resulting in increased mitochondrial fission. Reduced mitochondrial interconnectivity reduces reactive oxygen species (ROS) generating and oxidative capacities, while facilitating turnover of damaged mitochondria. **10**: Central/somatic microtubule abundance is increased, stabilizing core structure. Central re-localization of intermediate filaments contributes to core stability as well. **11**: A more globular phenotype with reduced cortical F-actin dynamics and actomyosin contraction arrests synaptic firing in neurons and reduces cellular adenosine-5′-triphosphate (ATP) and guanosine-5′-triphosphate (GTP) consumption among cell types. Created with BioRender.com.

Here, we have presented an argument for a contribution of cytoskeletal structure to metabolic rate depression in anoxic animals, such as turtles and goldfish, which we refer to as “Cytoskeletal Arrest” (Figure 4). In combining the idea of Bickler and Buck [10,155], that calcium-mediated actin depolymerization can contribute to metabolic rate depression, and the model constructed by Hawrysh and Buck [161], wherein mitochondria regulate anoxic calcium release, we propose a possible means of linking cytoskeletal structure to mitochondrial environmental sensing. Such a mechanism could explain anoxic cell shrinkage in turtles. While we originally envisioned a generalized cytoskeletal arrest based on reducing actin and tubulin ATPase and GTPase activity, the literature has suggested a type of cytoskeletal rearrangement based on locally modified microtubule stability and downregulation of peripheral F-actin assembly. Any downregulation of F-actin is likely to influence ion channel anchorage and therefore, function. The structural dynamics of the cytoskeleton and mitochondria influence each other, and mitochondrial metabolism has regulatory influences on the cytoskeleton through such intermediates as calcium, ROS, and gasotransmitters. The energetic cost of cell structure has seldom been considered, but the overwintering behaviour of the western painted turtle, and other anoxia-tolerant animals, creates conditions that may reasonably be expected to select for a more energetically efficient structural architecture. The anoxic turtle model presents an opportunity to improve our understanding of both the cytoskeleton and its contributions to assorted cellular energetic demands.

## Data Availability

The only data were in the form of images. Therefore, all data provided in manuscript.
